# Remedies for Coryza

**Published:** 1886-10

**Authors:** 


### *REMEDIES FOR CORYZA.*

Roben recommends the use of the following powder by insufflation:

| | | GRAMS. | |
|---|---|---|---|
| ℞ | Powdered Menthol, - | 20 | gr. iii. |
| | Roasted Coffee, - - - | 5 | ʒi., gr. xv. |
| | White Sugar, - - | 5 | ʒi., gr. xv. |

M. Sig.:—Snuff.

The above mixture has the appearance of snuff and is easily employed. The following is also recommended:

| | | |
|---|---|---|
| ℞ | Hydrochlorate of Cocaine, - - - | gr. iss. |
| | Browned Coffee. | |
| | White Sugar, - - - - | āā ʒ iss. |

M. Sig.:—Snuff.

—*Gaz. Med. de Bordeaux.*

